# Rett Syndrome in Males: The Different Clinical Course in Two Brothers with the Same Microduplication MECP2 Xq28

**DOI:** 10.3390/ijerph16173075

**Published:** 2019-08-23

**Authors:** Maria Bernarda Pitzianti, Angelo Santamaria Palombo, Susanna Esposito, Augusto Pasini

**Affiliations:** 1 Division of Child Neuropsychiatry, Department of Neuroscience, University of Rome Tor Vergata, 00133 Rome, Italy; 2 Department of Child Neuropsychiatry, USL Umbria 2, 05100 Terni, Italy; 3 Paediatric Clinic, Department of Surgical and Biomedical Sciences, Università degli Studi di Perugia, 06129 Perugia, Italy

**Keywords:** intellectual disability, MECP2, microduplication MECP2 Xq28, neurodevelopmental disorder, Rett syndrome

## Abstract

Rett syndrome (RTT) is a neurodevelopmental disorder with a genetic basis that is associated with the mutation of the X-linked methyl-CpG binding protein 2 (MECP2) gene in approximately 90% of patients. RTT is characterized by a brief period of normal development followed by loss of acquired skills and evolution towards impairment of brain and motor functions and multi-organ dysfunction. Originally, RTT was considered lethal in males as it has an X-linked dominant inheritance. However, although this syndrome has a higher incidence in females, rare cases are also documented in males. Here, we describe the case of an 11-year-old male patient with a microduplication MECP2 Xq28. Our patient is currently living, while his older brother with the same mutation died at the age of 9 years. We showed that the role of MECP2 as an epigenetic modulator and the X-chromosome inactivation pattern can explain the lethal clinical form of the older brother with the same microduplication MECP2 Xq28 presented by our patient who is still alive. Given the limited case history of RTT in males, further studies are needed to better characterize this syndrome in males and consequently improve the currently available therapeutic strategies.

## 1. Introduction

Rett syndrome (RTT) is a neurodevelopmental disorder with a genetic basis that is associated with the mutation of the X-linked methyl-CpG binding protein 2 (MECP2) gene in approximately 90% of patients [1]; other mutations in the CDKL5 and FOXG1 genes are associated with 10% of RTT cases [2,3].

RTT is characterized by a brief period of normal development followed by loss of acquired skills and evolution towards impairment of brain and motor functions and multi-organ dysfunction with the onset of the disease between 6 and 18 months of age [4]. It progresses through four stages, causing severe intellectual disability, epileptic encephalopathy, head growth deceleration, loss of functional use of the hands with stereotypical hand movements (hands washing), severe language regression, behavioral anomalies with autism-like symptoms, coordination and gait anomalies, breathing difficulties, cardiac dysrhythmia, and bone anomalies (e.g., scoliosis) [4]. RTT is predominantly found in females with an incidence of 1:10,000–20,000 live births, but in the general population its estimate drops to 1:30,000 live births and rare cases are reported in males [5]. Originally, RTT was considered lethal in males as it has an X-link dominant inheritance. However, rare cases are also documented in males and male RTT patients manifest a range of symptoms, including severe encephalopathy, mental retardation, and dystonia/apraxia [5].

Here, we describe the case of an 11-year-old male patient with a microduplication MECP2 Xq28 who had an older brother with the same mutation that died at the age of 9 years. Based on the most recent scientific findings about the role of the MECP2 gene, we have tried to explain the different clinical course of RTT in the two brothers.

## 2. Case Presentation

Our male patient was born at term after a normal pregnancy, but it was necessary to perform an emergency cesarean section for the appearance of acute fetal distress. Apgar score was 6 at 1 min, 8 at 5 min, and 9 at 10 min. His birth weight was 3130 g (75th–90th centile), his length 51 cm (50th–75th centile), and head circumference 31 cm (<3rd centile).

At birth, he presented bradycardia, asphyxia, generalized hypotonia, multi-organ dysfunction, and disseminated intravascular coagulation. On the first day, he presented with a post-asphyxiated seizure and was treated with phenobarbital for 6 months at the dose of 30 mg/kg/day. Subsequently, he developed epileptic encephalopathy with spasms similar to those of West syndrome and was treated with valproic acid (dose of 200 mg/kg/day), clonazepam (dose of 1.5 mg/kg/day), and levetiracetam (dose of 175 mg/kg/day). Electroencephalography (EEG) has always shown disorganized basal electrical activity and polymorphic and plurifocal anomalies (e.g., spikes, spikes-waves, slow waves).

Our patient showed a psychomotor development delay with poor and/or absent spontaneous motility, hypotonia of the trunk, and hypertonia of the four limbs. In the first year of life, brain ultrasound scans showed widespread cerebral atrophy and multicystic encephalopathy. Starting from the first year of life, he presented a severe psychomotor delay, bilateral horizontal nystagmus, dysphagia, gastroesophageal reflux, episodes of respiratory failure secondary to recurrent respiratory infections, and episodes of upper airway obstruction secondary to adenoid hypertrophy, which were treated with adenoidectomy. At the age of 5 years, he developed convex right scoliosis of the dorsolumbar tract with a left posterior hump. Currently, our patient presents facial dysmorphisms, spastic-dystonic tetraparesis, severe intellectual disability, lack of language and polymorphic epileptic seizures with varying frequency from one to ten a day, and constipation. Moreover, he had growth delay in weight (10° centile) and height (10° centile), failure to acquire head and trunk control, standing and autonomous walking, and presents clonic-tonic seizures with appearance during the sleep phase. Finally, he appeared soporous and for this reason it was not possible to subject him to the administration of psychometric scales.

In 2016, structural magnetic resonance imaging (MRI) showed large areas of gliosis in the occipital, frontal, and parietal brain regions bilaterally, widening of the periencephalic liquor spaces, and evident microcephaly (<3° centile). In the same year, electrocardiogram (ECG) showed a right branch block and computerized axial chest tomography showed bronchiectasis secondary to the previous respiratory infections.

RTT was suspected and array-CGH was performed, demonstrating the presence of a MECP2 gene microduplication (MECP2 Xq28). In our patient, genetic analysis was carried out on DNA extracted from peripheral blood. Through the analysis of 180,000 oligonucleotides at a medium resolution of 75 kb, a microduplication (extended approximately 464 kb) was found on the long arm of the X-chromosome in the Xq28 region. The microduplication, confirmed by molecular cytogenetic analysis (FISH), involves the MECP2 gene.

The older brother of our patient, born from term pregnancy, had the same microduplication in the Xq28 region, as detected by array-CGH. He died at the age of 9 years for paralytic ileus. Before his death, he presented a psychomotor delay, lack of language acquisition, motor stereotypy (e.g., swinging and hands washing), and poor and/or absent interest to the environment. Moreover, he presented a multisystem impairment, including inferior esophageal sphincter incontinence with gastroesophageal reflux, thymic hypertrophy, adenoid hypertrophy, laryngomalacia/tracheomalacia, and recurrent bronchopneumonic infections with secondary respiratory distress. He developed tonic-clonic seizures at the age of 8 years and was treated with valproic acid. No family history of neurologic disorders on the maternal side of other affected males was reported.

Clinical information and blood samples were obtained after approval from the Ethics Committee of the Umbria Region (PED-2018-31) and written informed consent was obtained from both parents. The parents also signed the consent for the publication of this case report.

## 3. Discussion

MECP2 has two functional domains, a methyl CpG binding domain and a transcriptional repression domain [6]. Scientific research has shown that mutations in the methyl CpG binding domain are associated with RTT [7]. In the past, it was believed that the MECP2 mutation gene was lethal in males [8]; subsequently, mutations of this gene have been documented in males and are associated with a wide variety of clinical phenotypes, including precociously fatal forms, congenital encephalopathy, severe intellectual disability, and classical RTT [9]. MECP2 controls gene expression and activation/repression of transcription, modulates chromatin architecture through binding to methylated DNA, and promotes genomic imprinting [10,11]. MECP2 expression is ubiquitous in humans, with higher levels of expression in the brain, lung, and spleen than those in the liver, heart, kidney, and intestines [12]. MECP2 deficiency or lack in the brain is responsible for neurological symptoms, as it causes failure to mature and maintain synapses in the central nervous system (CNS) [13]. Non-neurological symptoms, such as breathing difficulties, cardiac dysrhythmia, bone problems, and difficulty in feeding and limb movements, indicate the importance of MECP2 expression outside the CNS [14]. MECP2 expression in many organs besides the brain can explain the clinical form of our patient characterized by serious CNS abnormalities and multi-organ dysfunctions.

Scientific research has shown that MECP2 is involved in the control of epigenetic mechanisms responsible for the greater or lesser severity of the RTT [15]. These mechanisms include DNA methylation, histone post-translational modification, and noncoding RNAs [15] and are important for embryonic development and stem cell differentiation [16]. Moreover, since MECP2 is located on the X-chromosome, its inactivation in females has also been shown to impact the RTT clinical severity [12].

Moreover, recent studies have revealed that the genomes of even monozygotic twins are not similar due to the occurrence of somatic mutations. Somatic mutations in genes such as SCNA1 and ATP2A2 were detected in the peripheral tissues of one of the co-twins and not the other [13,14]. This correlated with only one of the twins showing disease pathologies. There have also been genomic studies detailing the occurrence of differential mutations within different tissues of the same individuals. Our case report discusses two brothers who are not identical twins and as such, there is a high probability that the differences in their genomes account for the differing lifespans regardless of the role of MECP2. In further studies, it would be useful to know the relative levels of MECP2 expression from the patient samples. In our patients it is possible that MECP2 levels were higher in the older brother accounting for the shortened lifespan. In this framework, we demonstrate that the role of MECP2 as an epigenetic modulator and the X-chromosome inactivation pattern can explain the lethal clinical form of the older brother with the same microduplication MECP2 Xq28 presented by our patient who is still alive.

## 4. Conclusions

RTT is a neurodevelopmental disorder most frequently observed in females. Here, we recorded the case of an 11-year-old male patient with RTT with a microduplication MECP2 Xq28 who had an older brother with the same mutation that died at the age of 9 years. Interestingly, MECP2 appeared as an epigenetic modulator. Given the limited case history of RTT in males, further studies are needed to better characterize this syndrome in males and consequently improve the currently available therapeutic strategies.
